# The South China Sea and Its Neglected Tropical Diseases

**DOI:** 10.1371/journal.pntd.0004395

**Published:** 2016-03-31

**Authors:** Peter J. Hotez

**Affiliations:** 1 Sabin Vaccine Institute and Texas Children’s Hospital Center for Vaccine Development, National School of Tropical Medicine, Baylor College of Medicine, Houston, Texas, United States of America; 2 James A Baker III Institute for Public Policy, Rice University, Houston, Texas, United States of America; 3 Department of Biology, Baylor University, Waco, Texas, United States of America; University of Washington, UNITED STATES

"The Falklands thing was a fight between two bald men over a comb." ‒ Jorge Luis Borges

**Fig 1:**
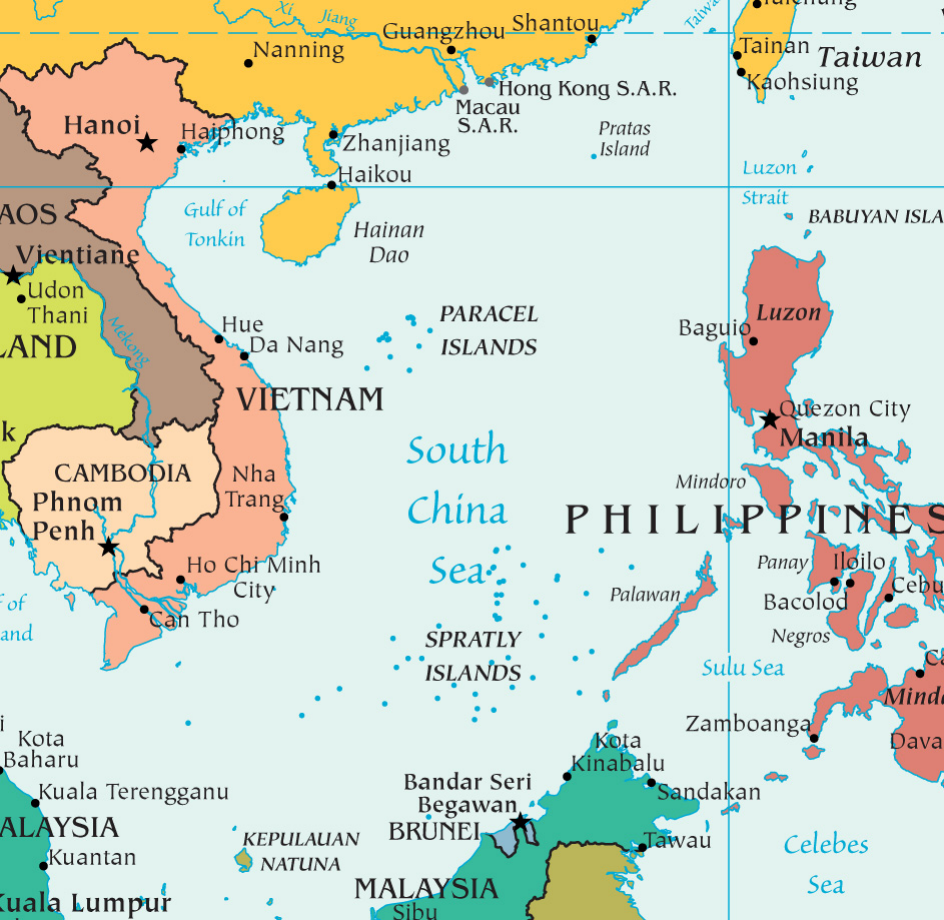
The South China Sea. Map modified from http://www.lib.utexas.edu/maps/middle_east_and_asia/southeast_asia_pol_2013.pdf, available at University of Texas Perry-Castañeda Library Map Collection: http://www.lib.utexas.edu/maps/asia.html.

The international maritime disputes in the South China Sea have now escalated following China’s construction of man-made islands and a new United States Naval presence that includes a guided missile destroyer. However, territorial claims between the nations surrounding these waters are not new. As both a major global shipping lane (it is considered one of the most trafficked sea lanes in the world) and host to significant oil and energy reserves, the numerous archipelago islands, straits, and shoals that comprise the sea (Fig 1) have been hotly contested for decades.

Each of the eight nations that border on the South China Sea—Brunei, China, Indonesia, Malaysia, the Philippines, Singapore, Taiwan, and Vietnam—has a major strategic interest in these latest foreign policy twists. Together, these countries are home to almost 2 billion people and have a total gross domestic product that exceeds US$10 trillion [1,2]. However, the economic strengths of these Asian nations are simultaneously sapped by hidden burden of poverty-promoting diseases known as the neglected tropical diseases (NTDs).

Shown in Table 1 is a summary of the major chronic and debilitating parasitic helminth infections affecting the eight South China Sea nations, based on the latest World Health Organization (WHO) preventive chemotherapy and transmission control data [3–5]. Altogether, at least 200 million people require regular and periodic treatments for their intestinal helminth infections, schistosomiasis, and lymphatic filariasis (LF), with Indonesia and the Philippines representing the most affected countries. Together, these helminth infections represent major reasons why groups of people in Asia cannot escape poverty. Intestinal helminth infections and schistosomiasis prevent children from growing to their full developmental potential and economic productivity, while LF renders adults too sick to lead productive and vigorous livelihoods [6]. A national “worm index” based on these metrics was recently published and was shown to be inversely related to a country’s human development index [6]. It was found that every South China Sea nation except for Singapore (or possibly Taiwan) has a positive worm index, while Indonesia and the Philippines exhibit worm indices that rival those found in sub-Saharan Africa and other less developed regions of the world.

**Table 1:** Parasitic helminth infections in the eight nations surrounding the South China Sea.

Country	Population [1,2]	School-aged children requiring treatment for intestinal helminth infections in 2014 [3]	School-aged children requiring treatment for schistosomiasis in 2013 [4]	Population requiring treatment for lymphatic filariasis in 2014 [5]	Total population requiring anthelmintic treatment^a^	Worm index^b^
People’s Republic of China	1,393,783,836	18,658,992	121,607	None	18,780,599	0.013
Republic of China (Taiwan)	23,434,000	Not reported	Not reported	Not reported	Not reported	Not reported
Philippines	100,096,496	19,255,752	508,621	21,879,837	41,644,210	0.416
Malaysia	30,187,896	No preventive chemotherapy required	None	176,748	176,748	0.006
Brunei	423,205	No preventive chemotherapy required	None	9,239	9,239	0.022
Indonesia	252,812,245	39,041,065	3,035	92,760,478	131,804,578	0.521
Singapore	5,517,102	None	None	None	None	0
Vietnam	92,547,959	4,580,664	None	Under surveillance	4,580,664	0.049
Total	1.9 billion	81.6 million	0.6 million	114.9 million	197.1 million	0.104

^a^ Result obtained by adding the numbers in columns 3, 4, and 5.

^b^ Result obtained as described in ref 6, i.e., dividing the total worms in column 6, by the population in column 2.

A major implication of these findings is that while nations fight for dominance over territories in the South China Sea, NTDs such as the major helminth infections that comprise the worm index actually represent the major threats to their economy and security.

Looking at these findings in a positive light, the high endemicity of helminth infections and other NTDs in the region creates new opportunities for international scientific and technical cooperation. Together, the eight South China Sea nations have sufficient economic clout and technical capacities in order to join forces and provide cooperative programs of mass drug administration. Such actions could lead to the regional elimination of LF, while having a substantial impact on intestinal helminth infections and schistosomiasis. Most of these nations also host a relatively sophisticated biotechnology infrastructure, including capacity for producing vaccines. Through joint scientific collaborations in programs of “vaccine diplomacy,” it should be possible to produce new generation NTD vaccines for many of the endemic diseases across the region [7].

Financial resources being devoted to maritime security in the region are escalating. According to one source, the US Government will spend US$425 million over the next five years [8], almost equivalent to its entire current budget for NTD mass drug administration. Similarly, it is likely the funds China now spends for thousands of acres in land reclamation to create islands in the South China Sea [9] likely exceeds its NTD budget. Rather than escalating tensions in the South China Sea and “fight over a comb,” to paraphrase Borges, there is an opportunity to work together for joint scientific cooperation as a means to lower national worm indices and ultimately reduce poverty and disease in East Asia.
